# Evolutionary History and Taxonomic Reappraisal of Coral Reef Rabbitfishes (Siganidae): Patterns of Lineage Diversification and Speciation

**DOI:** 10.3390/biology10111109

**Published:** 2021-10-28

**Authors:** Siti Zulaiha Zolkaply, Thinh Dinh Do, Md Asaduzzaman, Ying Giat Seah, David Hurwood, Peter Mather, Md Moshiur Rahman, Li Lian Wong

**Affiliations:** 1 Institute of Marine Biotechnology, Universiti Malaysia Terengganu, Kuala Terengganu 21030, Malaysia; zulaihazolkaply@gmail.com; 2 Faculty of Fisheries and Food Science, Universiti Malaysia Terengganu, Kuala Terengganu 21030, Malaysia; ygseah@umt.edu.my; 3 Institute of Marine Environment and Resources, Vietnam Academy of Science and Technology, Haiphong 04000, Vietnam; deepblue.th@gmail.com; 4 Department of Marine Bioresource Science, Faculty of Fisheries, Chattogram Veterinary and Animal Sciences University, Khulshi, Chattogram 4225, Bangladesh; asaduzzaman@g.ecc.u-tokyo.ac.jp; 5 Department of Aquatic Bioscience, The University of Tokyo, 1-1-1 Yayoi, Bunkyo-ku, Tokyo 113-8657, Japan; 6 South China Sea Repository and Reference Centre, Institute Oceanography and Environment, Universiti Malaysia Terengganu, Kuala Terengganu 21030, Malaysia; 7 Faculty of Science, Queensland University of Technology, Brisbane, QLD 4001, Australia; d.hurwood@qut.edu.au; 8 Australian Rivers Institute, Griffith University, Nathan, QLD 4111, Australia; p.mather1954@gmail.com; 9 Fisheries and Marine Resource Technology Discipline, Khulna University, Khulna 9208, Bangladesh; mrahmankufmrt@gmail.com; 10 UMT-OUC Joint Academic Center for Marine Studies, Universiti Malaysia Terengganu, Kuala Terengganu 21030, Malaysia

**Keywords:** rabbitfish, cytochrome oxidase I (COI), nuclear rhodopsin retrogene (RHO), nuclear DNA, morphology, phylogenetic, molecular clock

## Abstract

**Simple Summary:**

Herbivorous fish are recognized as being ecologically important to the health and survival of coral reef ecosystems because they remove algal turfs growing on corals. Apart from being one of the major components of herbivorous fish communities, rabbitfish are also characterized by possessing rabbit-like mouths. A total of 29 species of rabbitfish are confined to a single genus, *Siganus*, fish that are highly sought after for the aquarium trade and for food by humans. Natural hybridization between some species that have parapatric distributions across the Indo-West Pacific region may have homogenized their genotypic and morphological features. Relatively little is known, however, about how environmental factors may affect phylogenetic relationships among these siganid species. Based on sequencing of eight siganid species collected from the South China Sea and meta-analysis of sequences from ten siganid species retrieved from the NCBI database, we applied an integrated morphological–molecular approach to elucidate phylogenetic relationships and demographic histories of these species. Our results highlight that diversification and speciation of siganid species were influenced by a series of paleo-climatic events, changes to natural geographical distributions, and associated environmental changes. The target species were differentiated by body shape, and two morphometric parameters, notably body depth and snout length. Our results provide considerable baseline knowledge for strategizing improvement of both breeding and conservation programs for rabbitfish.

**Abstract:**

Rabbitfish (Siganidae) are coral reef fish that are distributed across diverse habitats that include estuaries, mangroves, reefs, and even seaweed mats. Given their ecological diversity and natural widespread distributions across the Indo-Pacific region, we were interested to investigate the evolutionary history of this group and patterns of divergence that have contributed to their present-day distributions. In the present study, samples were collected from the South China Sea to study taxonomic and phylogenetic relationships, and divergence times. We investigated the taxonomic relationships among modern rabbitfish species, reconstructed their molecular phylogeny, and estimated divergence times among selected lineages based on a fragment of the mtDNA cytochrome oxidase I (COI) and sequences of the nuclear rhodopsin retrogene (RHO). Our results indicate that modern rabbitfish likely originated in the Indo-West Pacific during the late Eocene [37.4 million years ago (mya)], following which they diverged into three major clades during the Pliocene/Pleistocene. Subsequent diversification and origins of the majority of siganids may likely be associated with episodes of paleo-oceanographic events, including greenhouse and glaciation events (Eocene–Miocene) as well as major plate tectonic events (Pliocene–Pleistocene). Some modern siganid species may naturally hybridize with congeneric species where their geographical ranges overlap. A comprehensive taxonomic analysis revealed that the phylogeny of Siganidae (cladogenesis of Clades I, II, and III) is characterized by divergence in several external morphological characters and morphometric parameters. Our study demonstrates that morphological characteristics, geographical heterogeneity, and environmental change have contributed to siganids’ historical diversification.

## 1. Introduction

Rabbitfish (Siganidae) comprise 29 nominal extant species that are confined to a single genus, *Siganus* [1,2,3]. These coral reef fish are widely distributed across the Indo-West Pacific region and inhabit a variety of habitat types, ranging from estuaries, mangroves, and seaweed mats to the reef front and reef flat [1]. Remarkable uniformity in morphological and morphometric characters that include number of fin spines and rays, lateral-line scales and gill-raker counts, tooth shape and tooth count have rendered species diagnoses of siganids extremely challenging [1]. Therefore, the current taxonomic classification of siganids relies largely on external phenotypic and morphometric characters that include coloration, body markings, snout shape, body depth, and shape of the caudal fin [1,2]. Siganids have also been categorized into “deep-bodied” and “slender-bodied” groups based on variation in standard length to body depth ratio [1]. Notably, intermediate body markings have been observed in some sister species that are distributed parapatrically in the Indo-West Pacific region. This suggests that introgressive hybridization may have occurred in the past, further affecting the taxonomic identification of siganids [3,4,5,6,7,8].

Phylogenetic studies have been conducted on siganid species collected from a variety of geographical localities using a variety of markers including allozyme polymorphisms [9], mitochondrial cytochrome b, and 16S rRNA genes [10,11,12,13], as well as a combined set of mitochondrial cytochrome b and nuclear ITS1 genes [7]. In a previous study by Lacson and Nelson [9], phylogenetic relationships were inferred from allozyme distances between 10 siganid species. Borsa et al. [10] explored the correlation between patterns of lineage diversification of Siganidae and ecological specialization and reported evolutionary relationships between parapatrically distributed sister species. Lemer et al. [11] barcoded 16 siganid species using the cytochrome b gene and assessed the correlation of quantified genetic divergence among populations between eight sibling taxa with their corresponding natural geographical distributions. Phylogenetic analyses based on mitochondrial and nuclear genes of 19 siganids by Kuriiwa et al. [7] identified natural hybridization between four pairs of closely related species and one pair of distantly related species within the Western Pacific region. Siqueira et al. [12] estimated divergence times for herbivorous coral reef species that included siganids, although only limited information related to historical speciation events that have specifically shaped the current morphological and biological traits of siganids were discussed in this study.

In the present study, we undertook an exhaustive taxonomic analysis of siganid species native to the Indo-Malay region to explore the potential for defining diagnostic characteristics capable of resolving existing taxonomic uncertainties. Considering that higher taxon numbers and the simultaneous use of genes with varying evolutionary rates and genomic positions may increase the accuracy of phylogenetic reconstructions [14,15], we reconstructed the siganid phylogeny using a new combination of gene sequences that consisted of mtDNA cytochrome subunit I (COI) and the nuclear DNA rhodopsin retrogene (RHO) for nominal Malaysian species and all known siganid species available in the public database, and validated our data with those of previous studies. The COI gene, a maternally inherited and haploid universal marker in general, provides sufficient sequence variation to allow species discrimination in most teleost fishes [16,17]. Despite having rarely been used as a nuclear gene marker in phylogenetic studies, the intron-less rhodopsin retrogene (RHO) that is associated with visual adaptations in aquatic species in dim light environments may be a quantitatively equivalent nuclear DNA marker for species identification and possibly for unravelling mechanisms of species diversification at the molecular level [18]. Furthermore, accurately estimating evolutionary timescales from molecular clock dating is fundamental to understanding evolutionary processes. Downstream biological and ecological studies of this herbivorous genus, which include biogeographical patterns of species diversity, ecological diversification, and evolutionary events, require knowledge of inferred time of divergence. Therefore, our study attempted to reconstruct and review phylogenetic relationships to assess: (1) morphological diversification and extent of genetic differentiation among siganids; (2) phylogenetic relationships of siganids based on the mtDNA (COI) gene and nuclear gene (RHO) sequences; and (3) to estimate divergence times among species, relevant for exploring historical patterns of their distribution and diversification.

## 2. Materials and Methods

### 2.1. Sampling

A total of 117 specimens from 8 siganid species were collected by local fishermen using commercial fish traps across the Terengganu coastal zones (Setiu Wetland, Pulau Kambing, Chendering, Marang, Dungun, and Kemaman) in the South China Sea off peninsular northeast Malaysia. Tissue samples were obtained from dead specimens, and no experiments on live animals were performed in this study. Fixation, photographic, and preservation methods of all fish specimens collected for the study generally followed procedures adopted by Seah et al. [19]. Dorsal fin samples were taken from all sampled fish and preserved in 95% ethanol for later molecular analysis. DNA samples and whole specimens (specimen ID: F1–F49, S1–S24, T1–T31, Dun1–Dun13) were deposited in the South China Sea Repository and Reference Center, Universiti Malaysia Terengganu as voucher specimens. Details of sample sizes, diagnostic morphological features, morphometric parameters, and GenBank accession numbers of all specimens used in the study are provided in Table S1.

### 2.2. Taxonomic Identification and Morphometric Analyses

Twenty-seven body landmarks and thirty morphometric measurements that can be used to infer individual body shape were collected on each sample specimen and are detailed in Figure S1. Morphometric measurements were conducted following Hubbs and Lagler [20]; all measurements were standardized using standard length to remove a size effect following Arjunaidi et al. [21] for morphometric parameters/standard length. Morphological descriptions were performed according to modified protocols of Seah et al. [19]. Species identifications were determined following Woodland [1].

R version 3.6.1 [22] was used for performing cluster analyses of morphometric variation among sampled siganid species. All morphometric data were tested for normality using the Shapiro–Wilk method [23] and data were log-transformed to normalize where required. The “car” package [24] was used in the univariate analysis of variance (ANOVA) and a post hoc Tukey multiple comparison tests was completed for each morphometric character using the “multcomp” package [25]. The ANOVA model showed that all morphometric distances differed significantly, to varying degrees, among sampled siganid species. As a result, all morphometric distances were used to obtain consistent outcomes from the multivariate analyses. To confirm the existence of morphometric variation among the sampled siganid species, a number of different multivariate approaches that included principal component analysis (PCA), canonical variates analysis (CVA), and cluster analysis (CA) using a Euclidean distance method were employed here. PCAs were executed using the ‘FactoMineR’ package [26]. CVA was performed in the ‘MASS’ package [27]. Euclidean distance as a measure of dissimilarity and UPGMA (unweighted pair group method with arithmetical average) as the clustering algorithm was adopted in the cluster analysis. Cluster analysis was undertaken using the ‘dendextend’ package [28].

Discriminant analysis was also performed using stepwise statistics to identify the best morphometric characters that could significantly discriminate among the three deep-bodied siganid species. This analysis used stepwise statistics to determine the morphometric characters that best differentiate the three deep-bodied species: *S. guttatus*, *S. javus*, and *S. stellatus*. The same analysis was not performed on slender-bodied siganid species, given that no significant differences were evident among them for all characters except for total individual length. This analysis was conducted using SPSS version 22 software (IBM Corp, Armonk, NY, USA).

Differences among deep-bodied species [*S. guttatus* (*n* = 14), *S. javus* (*n* = 20) and *S. stellatus* (*n* = 20)] were investigated by applying the non-parametric Kruskal–Wallis test to identify the most significant parameters that could discriminate among deep-bodied species. Results of the Kruskal–Wallis test of pairwise comparisons for deep-bodied species are summarized in Figure S2. Moreover, PCA was also performed to determine which morphometric variables were mostly variable among the three deep-bodied siganid species. Based on the outcomes of these approaches, significant morphometric variables were plotted against standard length to investigate visual differences among the three deep-bodied species. All the plots were made using the ‘ggplot2’ package [29].

### 2.3. DNA Extraction, Amplification and Sequencing

Total genomic DNA was extracted from dorsal finclips using a GF-1 Tissue DNA Extraction Kit, following the manufacturer’s protocol (Vivantis, Shah Alam, Malaysia). mtDNA COI and the nuclear rhodopsin retrogene (RHO) were amplified with primers and PCR conditions which are detailed in Table S2. PCRs were performed in 25 µL final volumes containing 1× PCR buffer, 1.25 mM MgCl_2_, 0.2 mM dNTPs, 0.4 µM forward and reverse primers, 0.1 U of Taq polymerase, and 100 ng of DNA. The thermocycling profile for the COI gene was as follows: initial denaturation for 4 min at 95 °C; 30 cycles of denaturation at 95 °C for 30 s, annealing at 51.3 °C for 30 s, and extension at 72 °C for 1 min; and final elongation at 72 °C for 7 min. The rhodopsin gene was amplified using the following PCR settings: 95 °C for 4 min; 40 cycles of 30 s at 94 °C, 30 s at 60 °C, and 45 s at 72 °C; and 72 °C for 8 min and 10 s. Reactions were performed in a thermocycler. Amplified products were purified using a QIAquick PCR purification kit (Qiagen, Hilden, Germany), and sequenced bi-directionally using the BigDye^®®^ Terminator v3.1 cycle sequencing kit (Applied Biosystems, Foster City, CA, USA). Sequencing reactions were conducted in a 96-capillary 3730*xl* DNA Analyzer (Applied Biosystems, Foster City, CA, USA). Sequences were deposited in GenBank with accession numbers listed in Table S1.

### 2.4. Phylogenetic Analysis

Forward and reverse sequences were aligned to generate contigs using BioEdit Sequence Alignment Editor v 7.0.1 [30]. Additional GenBank and BOLD sequence data from ten additional siganid species were obtained to increase total taxonomic coverage for the phylogenetic analysis (Table S3). In total, 156 sequences from 18 of the 29 currently described siganid species were used for phylogenetic reconstruction. In addition, several acanthurid species (family Acanthuridae) were selected as outgroups for the analysis based on previously published work [31]. Consensus sequences for each species were also generated from contigs and sequences from databases, and all were aligned using CLUSTALW [32]. The number of haplotypes and polymorphic sites, haplotype diversity (Hd), and nucleotide diversity (π) were estimated in DnaSP v 5.10.01 [33]. We conducted separate and combined phylogenetic analyses for both gene datasets to avoid the possibility of erroneous species trees generated from the maternally inherited COI gene dataset alone [34]. Prior to concatenation of the two gene datasets, an incongruence length difference (ILD) test [35] was performed in PAUP [36]. A supermatrix was generated by concatenating both COI and rhodopsin genes for eight sampled species using MESQUITE v 3.2 [37]. The concatenated dataset was used to validate our phylogenetic analysis, given that combined analysis of the nuclear and mitochondrial genes has been reported to produce well-resolved phylogenetic relationships [38,39,40]. Longer sequences helped to reduce the stochastic error of substitutions across sites, because they complement each other to increase bootstrap support for nodes in the phylogenetic tree [41]. The most appropriate nucleotide substitution models for all phylogenetic trees were determined by applying Akaike Information Criterion (AIC) scores, and lowest Bayesian Information Criterion (BIC) values using a best-fit substitution model analysis estimated in MEGA v 7.0. From this analysis, HKY + G was selected as the best-fit substitution model [42]. Pairwise genetic distance estimates between species pairs for both separated and concatenated sequences were based on the HKY + G model [43], and details of nucleotide substitutions for each gene are detailed in Tables S4 and S5. Gene trees were constructed for 18 siganid taxa and 4 acanthurid outgroup taxa using COI, RHO, and concatenated datasets for both genes (only 8 siganids) based on Maximum Likelihood (ML) algorithms using MEGA v 7.0 [42]. We applied HKY + G as the evolutionary model in both trees, with all positions containing gaps or missing data removed. The reliability of each clade identified in the analysis was evaluated using 10,000 bootstrap replicates. Rate variation among sites was modeled via a gamma distribution for both trees. For the ML tree reconstruction, the initial tree(s) for the heuristic search were obtained automatically by applying Neighbor-Joining and BioNJ algorithms to a matrix of pairwise distances estimated using the Maximum Composite Likelihood (MCL) approach. This was followed by topology selection applying a superior log-likelihood value.

### 2.5. Divergence Time Estimation and Phylogeny

A time-calibrated tree based on COI gene sequences was constructed in BEAST v 1.7.4 [44] using a random starting tree generated from the simplified dataset produced from a single consensus sequence generated for each sampled species. We adopted a Random Local Clock (RLC) model for the time tree estimation that was implemented using the Bayesian phylogenetic software, BEAST v 1.7.4 [44]. RLC is a model of evolutionary rate heterogeneity that is based on the assumption that closely related lineages share distinct substitution rates and the amount of evolutionary variance across a tree is between both models of a strict clock and a relaxed clock [45]. BEAST v 1.7.4 uses Bayesian inference and the Markov Chain Monte Carlo (MCMC) procedure to derive a posterior distribution of local rates and times, where priors (known divergence time for at least one node) are selected and specified for local clock time calibration [41,44,46,47]. In this study, we used two calibration points for the closest genus, *Acanthurus* spp. as the prior to date our tree, where the time of divergence for these species were estimated based on fossil-calibrated time trees in earlier studies [13,40]. Siquiera et al., 2019 [13] used published morphological cladograms of fossils and extant surgeonfish species from the genus *Acanthurus* to constrain the fossil positions. To further estimate the node ages for each focal taxa, genetic dataset and fossils tip dates were implemented in the fossilized birth–death (FBD) models [48]. In Sorenson et al., 2013 [40], two fossil calibration points for *Sorbinithurus sorbinii* (50 mya) and *Kushlukia permira* (55.8 mya) were used to date their time tree. Divergence time estimated from these studies [13,40] for the species pairs *Acanthurus chirurgus* vs. *A. tractus* (mean = 5.0 million years ago [mya], SD = 0.5) and *A. olivaceus* vs. *A. tennentii* (mean = 4.0 mya, SD = 0.5) were then used as the time of the most recent common ancestor (tMRCA) for the present study. Input files were generated using BEAUti v 1.7.4 [44] default settings for prior selection in the RLC model, applying a Yule speciation prior, an uncorrelated relaxed log-normal molecular clock, and the HKY substitution model. The obtained tMRCAs were then constrained in the analysis by the normal prior distribution range. Two independent runs were performed, each for 150 million generations, with trees sampled every 10,000 generations. The first 25% of trees were discarded as a burn-in. Each run was checked for appropriate mixing and convergence based on ESS values of >200 in Tracer v 1.6 [4,49]. A maximum clade credibility tree with a mean nodal height was generated using TreeAnnotator v 1.7.4 [44] before final manipulation of the time-calibrated phylogeny in FigTree v 1.4.2. [44].

## 3. Results

### 3.1. Morphological Assessment

**Figure 1:**
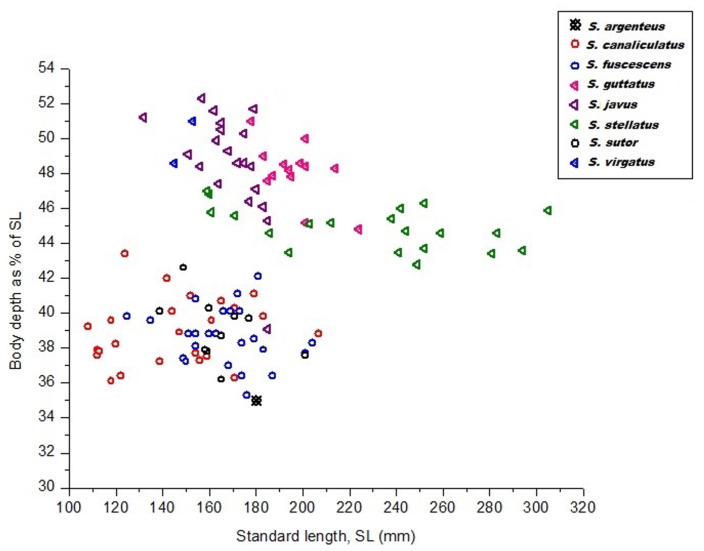
Body depth as a function of the standard length of eight siganid species collected from the South China Sea, Terengganu, Malaysia.

Eight siganid species were sampled for the study. Mean values for body depth and snout length of all deep-bodied species (*Siganus guttatus*, *S. javus*, *S. stellatus*, and *S. virgatus*) were significantly different from all slender-bodied species (*S. canaliculatus*, *S. fuscescens*, and *S. sutor*) and moderately slender-bodied species (*S. argenteus*) (*p* < 0.05). Table S1 contains details of measurements for three morphometric parameters from siganid species collected in Terengganu, Malaysia. Data illustrate that all deep-bodied species were brightly colored, whereas the remaining four species were drab in color. The deep-, slender-, and moderately slender-bodied species could be discriminated based on their body depth (Figure 1). Average body depth of deep-bodied species (*p* < 0.05) was greater than those of slender- or moderately slender-bodied species (Table S1 and Figure 1).

**Figure 2:**
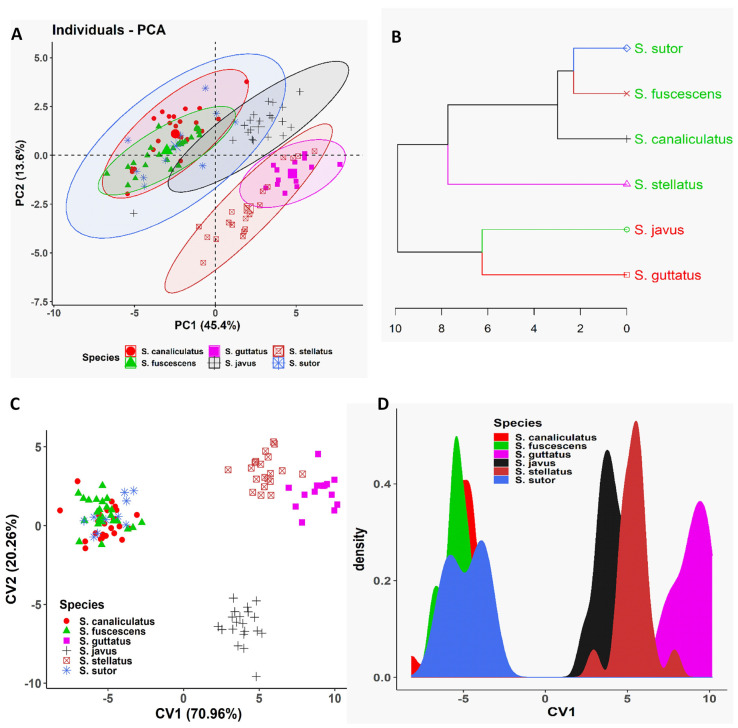
Multivariate analyses of morphometric data of different siganid species collected from the South China Sea, Terengganu, Malaysia. (**A**) Biplot of principal component analysis is shown based on the morphometric data of different siganid species. (**B**) Dendrogram derived from cluster analyses of morphometric measurements is shown based on Euclidean distance of different siganid species. (**C**) Biplot of canonical variates analysis (CVA) is shown based on the morphometric data of different siganid species. (**D**) Coordinate density plot of canonical variate 1 is shown based on the morphometric data of different siganid species.

To confirm the existence of morphometric variation among our siganid species, multivariate approaches including PCA, CVA, and CA using a Euclidean distance method were employed. Outcomes of the various multivariate analyses employed indicated that the morphometric divergence of our siganid species overlapped and only really distinguished between them by body depth, i.e., slender and deep-bodied species (Figure 2). The three slender-bodied species (*S. canaliculatus*, *S. fuscescens*, and *S. sutor*) were essentially not distinguishable based on this measure, whereas the three deep-bodied species (*S. stellatus*, *S. guttatus*, and *S. javus*) could be clearly discriminated from each other (Figure 2). For slender-bodied species, intermediate body markings have been reported in some closely related sister species including *S. guttatus*–*S. vermiculatus* and *S. fuscescens*–*S. canaliculatus*, indicative of overlapping morphometric traits due to potential past hybridization.

**Figure 3:**
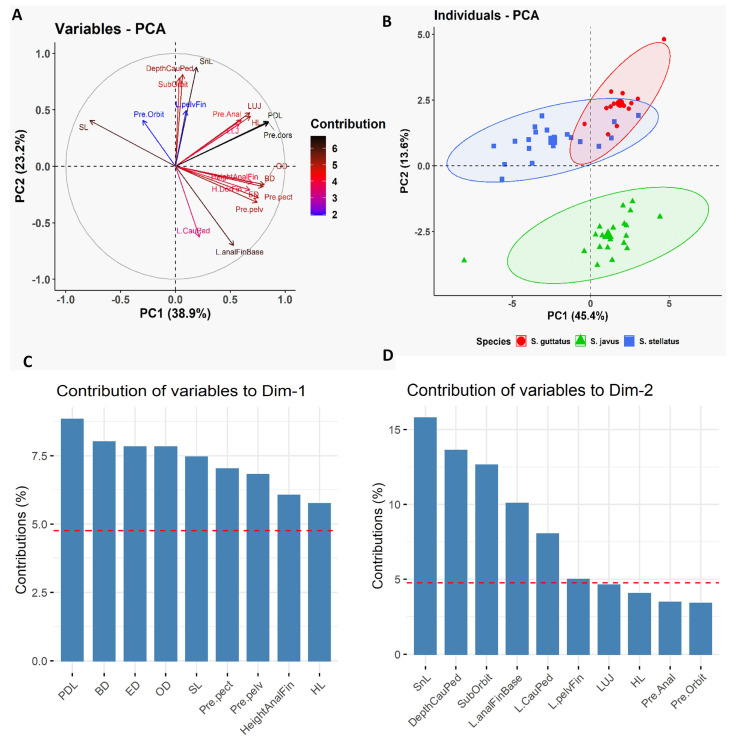
Principal Component Analysis (PCA) of morphometric data of three deep-bodied siganid species [*S. guttatus* (*n* = 14), *S. javus* (*n* = 20) and *S. stellatus* (*n* = 20)] collected from the South China Sea, Terengganu, Malaysia. (**A**) Biplot of the variable PCA is shown based on the morphometric data of three deep-bodied siganid species. (**B**) Biplot of the individual PCA is shown based on the morphometric data of three deep-bodied siganid species. (**C**) Relative contribution of morphometric variables to Dim-1 to discriminate three deep-bodied siganid species. (**D**) Relative contribution of morphometric variables to Dim-2 to discriminate three deep-bodied siganid species.

**Figure 4:**
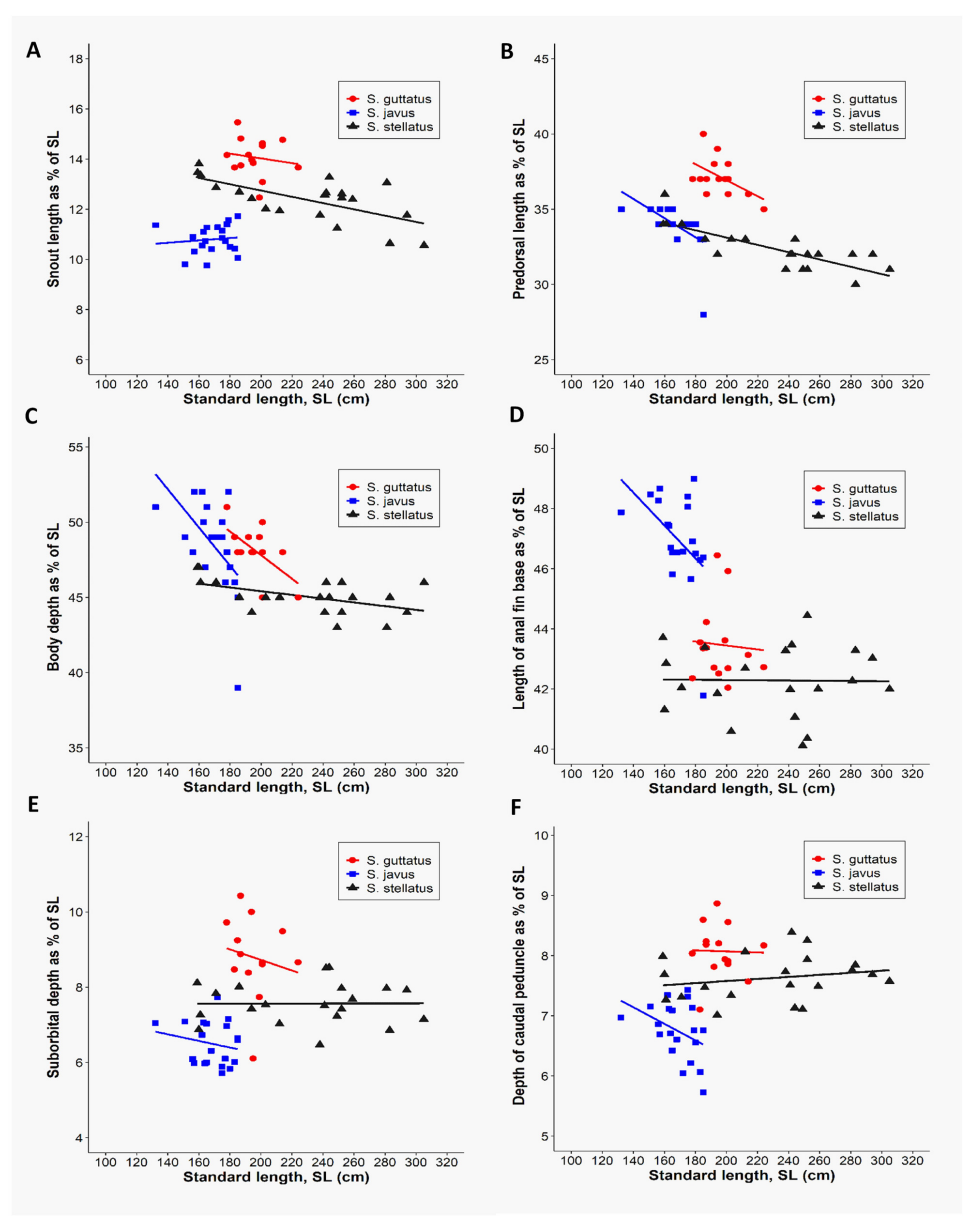
Discrimination of three deep-bodied siganid species [*S. guttatus*(*n* = 14), *S. javus* (*n* = 20), and *S. stellatus* (*n* = 20)] based on the six morphometric variables relative to the standard length of each species. (**A**) Relationship between snout lengths as a function of standard length. (**B**) Relationship between predorsal lengths as a function of standard length. (**C**) Relationship between body depths as a function of standard length. (**D**) Relationship between lengths of anal fin base as a function of standard length. (**E**) Relationship between suborbital depths as a function of standard length. (**F**) Relationship between depths of caudal peduncle as a function of standard length.

Furthermore, we employed PCA to determine which morphometric traits were most efficient at discriminating the three deep-bodied species (Figure 3). As reported earlier, the multivariate spaces of the three deep-bodied species were clearly different (Figure 3). PC1 (38.9% variability) and their differentiation was dominated by predorsal length (PDL) and body depth (BD), whereas PC2 (23.2% variability) was largely influenced by the snout length (SnL), depth of caudal peduncle, suborbital depth, and length of anal fin base (Figure 3). The three slender-bodied species (*S. canaliculatus*, *S. fuscescens*, and *S. sutor*) could not be distinguished based on any of morphological trait parameters, whereas deep-bodied species were distinguished successfully using the same six morphometric characters (Figure 4). Further classification was performed between individuals of the three deep-bodied species based on discriminant analysis applying a stepwise method and a Kruskal–Wallis test (*p* < 0.05). Results of pairwise comparisons between species are summarized in Figure S2. Our results show that snout length is the best parameter for discriminating among the three deep-bodied species (*S. stellatus*, *S. guttatus*, and *S. javus)* (Figure S2 and Table 1). Based on Table 1, *S. javus* (*p* = 0.683) displayed the lowest value for snout length as a percentage of standard length (9.8% to 11.7%) (SL: 130–185 mm), *S. stellatus* (*p* = 0.002) was 10.6% to 13.8% (SL: 155–310 mm) and *S. guttatus* (*p* = 0.604) was 12.5% to 15.5% (SL: 175–225 mm). Due to the presence of overlapping snout lengths, *S. guttatus* could be differentiated from *S. stellatus* and *S. javus* based on predorsal length (Figure 4B).

### 3.2. Haplotype Diversity and Sequence Divergence

A total of 99 COI (651 bp) and 101 rhodopsin (460 bp) gene fragments were sequenced successfully for the 8 siganid species sampled in Malaysian coastal waters (Table S1). From a total of 138 polymorphic sites, 120 were found to be parsimony-informative, whereas 18 were singleton variable sites across consensus COI haplotypes in the 8 siganid species examined here. The rhodopsin gene dataset comprised sequences from 8 siganid species, with 19 of 35 polymorphic sites being parsimony-informative. Both genes displayed high haplotype diversity (Hd) (COI = 0.965, rhodopsin = 0.853); in contrast, nucleotide diversity (π) (COI = 0.077; rhodopsin = 0.019) was relatively low. Of interest, a substantially large range of interspecific genetic distance estimates based on the concatenated gene dataset were evident among the siganid species sampled here, that ranged from 0.002 (*S. stellatus*/*S. punctatus* and *S. fuscescens*/*S. canaliculatus*) to 0.219 (*S. rivulatus*/*S. corallinus*). Similar trends were observed for the single gene datasets (Tables S4 and S5).

### 3.3. Phylogenetic Analyses

**Figure 5:**
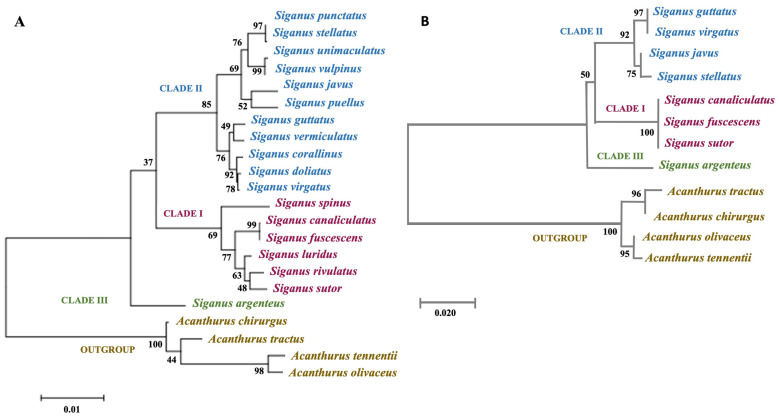
Maximum likelihood (ML) phylogenetic analyses of siganid taxa based on COI and RHO gene sequences. (**A**) ML phylogenetic relationship between 18 siganid taxa based on mitochondrial COI gene sequences, and (**B**) ML phylogenetic relationship between 8 siganid taxa based on RHO gene sequences. Bootstrap values are shown next to the branches, and the scale bar indicates the estimated number of substitutions per site. Clade I: slender-bodied, Clade II: deep-bodied, Clade III: moderately slender-bodied.

Our phylogenetic analyses are presented in Figure 5A (COI marker), Figure 5B (RHO marker), and Figure S3 (concatenated data). Maximum likelihood (ML) trees generated from the COI gene (Figure 5A) datasets showed that Siganidae contained three major species clusters (Clades I, II, and III): Clade I consisted of slender-bodied species (*S. canaliculatus*–*S. fuscescens*, *S. rivulatus*–*S. sutor*, *S. luridus*, and *S. spinus*); Clade III consisted of a single species, *S. argenteus*, that formed the moderately slender-bodied group; all deep-bodied species grouped in Clade II (*S. punctatus*–*S. stellatus*, *S. unimaculatus*–*S. vulpinus*, *S. javus*–*S. puellus*, *S. guttatus*–*S. vermiculatus*, *S. doliatus*–*S. virgatus* and *S. corallinus*). The position of *S. argenteus* was divergent from both slender and deep-bodied siganids in the ML tree (Figure 5A). Although only eight species were used to generate the ML tree based on the RHO gene (Figure 5B), the pattern of species diversification was similar to that grouped in Clade I (*S. canaliculatus*–*S. fuscescens*–*S. sutor*), deep-bodied species in Clade II (*S. guttatus–S. virgatus*, *S. javus*–*S. stellatus*) and moderately slender-bodied *S. argenteus* in Clade III. Phylogenetic analysis of concatenated gene sequences (Figure S3) yielded topologies that were congruent with topologies obtained from analyses of the independent gene datasets, and relationships among taxa were highly resolved with strong statistical support (bootstrap value: 99–100% probability value near to 1.0 or >0.8) except for nodes that connected *S. javus* and *S. stellatus* (bootstrap value: 89%).

### 3.4. Molecular Dating

**Figure 6:**
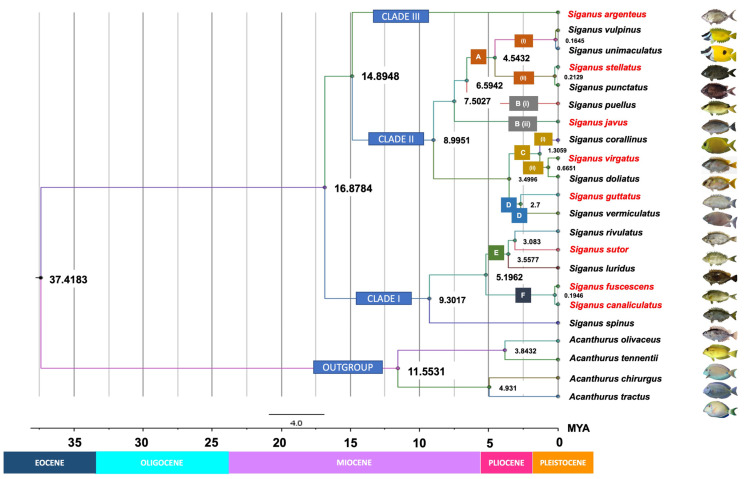
Time-calibrated phylogeny for *Siganus* species based on COI Bayesian inference. Numbers near the nodes are denoted as millions of years ago (mya), corresponding to the geologic periods. Text boxes detail the morphological or morphometric features unique to each clade and taxon. Clade I: slender-bodied, Clade II: deep-bodied, Clade III: moderately slender-bodied. A (i) = Head and body with no spots, snout strongly tubulated; A (ii) = Head and body completely covered with numerous light or dark spots, snout not tubulated; B (i) = Body compressed and not particularly deep, prominent black spots are present above the eyes within a dark diagonal band that extends from the chin to the nape; B (ii) = Body deep and compressed, no prominent black spots above the eyes. C = Have similar range of head depth at the edge of the pre-opercle as a percentage of the standard length; C (i) = No band is present on the head or body, snout is slightly pointed; C (ii) = Two dark bands are present from the chin to the nape and from the base of the pelvic fins to that of the dorsal fin, snout is blunt; D = Have similar habitat preference and ecological behavior: inhabits shallow coastal areas and river mouths and tolerate low salinity; schooling throughout life. E = No pearly spots on the head or body; F = Pearly blue spots are present on the head and body. Species or groups of species were classified based on morphological characteristics, morphometric measurements, habitat preferences, or ecological behavior. Names in red indicate species that were sampled in this study. BD = body depth; ED = eye diameter; Max. TL = maximum total length.

Our time-tree analysis (Figure 6) essentially recovered a similar topology to those retrieved in the ML analyses for COI gene sequences (Figure 5A). They differed only in the positions of *S. puellus* and *S. javus* that were fully resolved in the ML trees (Figure 5A). The time-tree analysis estimated an early Eocene crown age for modern siganids (37.4 mya). Time of diversification of the three clades, consisting of slender-bodied (Clade I), deep-bodied (Clade II), and moderately slender-bodied (Clade III) species, was estimated at 16.9 mya. This result suggests that the three clades originated in the late Miocene, with *S. argenteus* diverging from other deep-bodied species, approximately 14.9 mya. The crown for deep-bodied species originated in the late Miocene (about 9 mya), and the crown for slender-bodied species was estimated at around 9.3 mya. *S. javus* in the Indo-Pacific region had apparently split from remaining deep-bodied extant species in the late Miocene (7.5 mya). Two closely related species, *S. fuscescens* and *S. canaliculatus*, have apparently radiated only recently from a common ancestor in the late Pleistocene (approximately 195 kya). Of note, all phylogenetic analyses in our study, including our time-calibrated Bayesian phylogeny (Figure 6), consistently support species relationships uniquely characterized by a combination of body shape in addition to two morphometric parameters (body depth and snout length).

## 4. Discussion

### 4.1. Phylogenetic, Systematic, and Ecological Inferences

Our phylogenetic analyses based on the COI gene fragment (Figure 5A) clearly clustered siganids into three major clades: Clade I (*S. canaliculatus*, *S. fuscescens*, *S. sutor*, *S. rivulatus*, *S. luridus*, and *S. spinus*), Clade II (*S. punctatus*, *S. stellatus*, *S. unimaculatus*, *S. vulpinus*, *S. puellus*, *S. javus*, *S. guttatus*, *S. vermiculatus*, *S. corallinus*, *S. doliatus*, and *S. virgatus*), and Clade III (*S. argenteus*). The three groups align closely with those reported in earlier molecular studies [7,10]. Diversification of the 18 siganid species investigated here primarily involved changes to external morphological characteristics, morphometric measurements, and the use of habitat by individual taxa.

The morphological trait that most clearly aligned with the recognition of distinct taxa was body shape, whereas morphometric parameters including body depth and snout length were more useful in higher taxonomic comparisons. Clade III contains only a single species, *S. argenteus*, that possesses a moderately slender-bodied shape, the least body depth (35.0% SL), and the shortest snout length (8.8% SL). Species in Clade I were slender and compressed in body shape (body depths in the range of 36–40% SL) and had short snouts (<10.8% SL). Unlike those in Clades I and III, species in Clade II possessed a deep and compressed body shape, the greatest body depth (>40% SL), and a long snout (≥10.8% SL). Although some species in Clade I had blunt snouts, the average snout length of all individuals in this group was longer than those in the other two clades.

Morphological divergence of species in the three clades likely provides general functional phenotypes that are adapted to different niches in marine habitats. Small body depth and slender bodies of species in Clades I and III would allow the ability to disperse longer distances due to reduced body friction, whereas deep body shapes in Clade II species may facilitate mobility in more spatially complex habitats [50]. The drab body color of species in Clades I and III likely provides advantages for living in open habitats, including clear tropical and coastal waters. This generalized phenotype was also complemented by more blunt and shorter snouts that potentially facilitate feeding on floating algae, filamentous algae, seagrasses, and/or algae attached to rocks or corals [1]. In contrast, the presence of longer snouts in deep-bodied species (Clade II) likely facilitates advantages for foraging, particularly in more structurally complex habitats, including small crevices, rocky areas, coral substrates, and/or coarse benthic seaweeds [50,51,52]. As with the patterns of species divergence in COI gene-based ML phylogeny (Figure 5A), the RHO gene-based ML tree also categorized the same groups of species according to body proportion into three clades.

### 4.2. Biogeographic and Evolutionary History

Our time-calibrated tree (Figure 6) suggests that siganids diverged from their sister lineage, the acanthurids, approximately 37.4 mya during the late-Eocene and then underwent further diversification during the mid-Miocene to early Pleistocene. Overall, the phylogenetic pattern generated here is consistent with the divergence times estimated for siganids by Siqueira et al. [12,13] based on cytochrome b, 16S ribosomal RNA, and ITS1 gene fragments, except for the positioning of *S. argenteus*. Any minor differences in timing can be explained by use of different markers in our study (COI and RHO gene) from those used previously (cytochrome b, 16S ribosomal RNA and ITS1 genes) [53]. Timing of the origin of siganids coincided with the rapid proliferation of complex perciform lineages that constitute all modern reef fish during the Eocene [54,55,56]. Emergence of siganids with other reef-associated species, including surgeonfish, butterflyfish, and labrids [40,57,58], has been linked to the evolution and expansion of extensive tropical shallow-water reef environments that resulted from greenhouse impacts on climate across the Paleocene–Eocene thermal maximum. This process caused substantial increases in sea-surface temperatures and raised sea levels at low latitudes. In addition, cooling temperatures at the southern poles at approximately 37 mya may have influenced the biogeography of many reef fish species and prompted the adaptive radiation of siganids toward northeastern biogeographical regions where they are found today [55,59,60].

Although siganid fossils from the Eocene have been recorded in Italy, when the group potentially first evolved, fossil evidence has suggested that ancient species had wider habitat preferences and lacked the more specialized morphologies found in modern reef-adapted taxa such as those examined here [7,61,62]. The siganid phylogeny developed here raises the possibility that lineages investigated here potentially originated in the Indo-Malay region when ancestors of *S. argenteus* (and presumably *S. woodlandi*) [7,10] first evolved during the Miocene (14.9 mya). This hypothesis concords with results documented in previous studies [1,13,63]*. S. argenteus* is the only modern siganid species to possess a pelagic pre-juvenile stage, and it lays pelagic eggs as do modern acanthurid fishes [1]. We postulate that a fusiform-like body and the presence of a pelagic larval stage would provide greater capacity to disperse over large distances across a broad geographical region from the Indo-Malay region to the Red Sea westward, and even to the Pitcairn Island region eastward, when Asia–Pacific continents were historically connected via an inferred paleo-oceanic pathway [64]. We believe that constructing a phylogeny with *S. argenteus* samples from many different geographically separated regions could be useful for examining ocean history, biogeographical variance, and recent siganid speciation more widely across the Indo-Pacific region. Diversification leading to a variety of morphological modes that characterize *S. javus*, *S. puellus*, and *S. spinus* today potentially occurred during the Miocene epoch (6.6–9.3 mya). During this cooler period, glaciation increase resulted in significant changes to shallow-water marine habitats, sea temperatures, sea levels, and relative ocean productivity [59,65,66]. With raised sea levels, coral reef systems likely expanded, and algal-reef growth presumably provided more favorable habitats for algal-feeding species in shallow coral reef and coastal waters across a wide geographical distribution in the Indian, Indo-Malay, and Western Pacific oceans.

**Figure 7:**
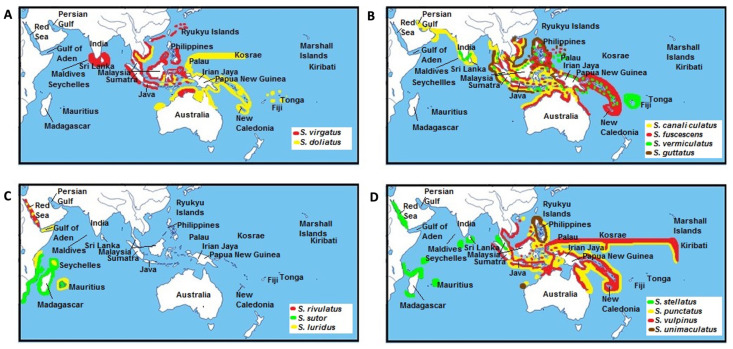
Distribution ranges of siganid sister species across the Indo-Pacific region adapted from Woodland [1]. Distribution regions for (**A**) *S. virgatus* and *S.doliatus*, (**B**) *S. canaliculatus*, *S. fuscescens*, *S. vermicualtus*, and *S. guttatus*, (**C**) *S. rivulatus*, *S. sutor*, and *S. luridus*, (**D**) *S. stellatus*, *S. punctatus*, *S. vulpinus*, and *S. unimaculatus*.

As with other marine fauna, more recent siganid diversification potentially occurred during periods of fluctuating sea levels during the Pliocene/Pleistocene epochs [67,68]. The emergence of the Sunda Shelf between the Indo-Malay region and northern Australia at this time could have facilitated the divergence of *S. virgatus* and *S. doliatus* (Figure 7A) [69]. Closely related sister species, including *S. guttatus*–*S. vermiculatus*, and *S. fuscescens*–*S. canaliculatus* may have developed overlapping geographical distributions at this time (Figure 7B). In parallel, *S. luridus* that now co-occurs with *S. sutor*–*S. rivulatus* in the Red Sea and the Western Indian Ocean (Figure 7C) is closely related to these two species [1]. In contrast, isolation from a common ancestor may have resulted in the divergence of *S. sutor*, a species endemic to the Western Indian Ocean, and *S. rivulatus*, which is native to the Red Sea [1]. Of interest, the phylogenetic position of *S. luridus* in our study is different from that in a previous study by Borsa et al. [10]. In Borsa’s study, *S. luridus* was placed in the first branch of the fusiform group and apparently diverged at an earlier time from other species in this group (Figure 5). Here, *S. luridus* was placed in the middle of the clades, which is sister to a branch comprising *S. rivulatus* and S*. siganus*.

This result is congruent with the analysis of divergence time (Figure 6) as well as the modern geographical distributions of these species. As mentioned above, *S. luridus* and *S. rivulatus* occur in the Red Sea and the Western Indian Ocean, whereas *S. spinus* and *S. fuscescens* are restricted to the Western Pacific Ocean [1]. Our phylogenetic tree and divergence times also support Borsa’s inference that *S. luridus* and *S. spinus* are not sibling species, even though their morphological traits are very similar [10]. For species with non-overlapping distributions, including *S. stellatus*/*S. punctatus* and *S. vulpinus*/*S. unimaculatus* (Figure 7D), diversification was potentially triggered by changes to sea levels during the Indonesian throughflow [70], or possibly by tectonic activity across the Indonesian region that changed surface water circulation patterns in the Indian and Pacific Oceans [56]. However, a study of correlations between sister-species relationships and their individual geographical distributions is now warranted to evaluate a divergence hypothesis.

### 4.3. Hypothesis: Natural Hybridization and Speciation

Hybridization between siganid sisters’ species is not uncommon, as previously noted by Kuriiwa et al. [7]. Although some ecological traits, including pairing and schooling behavior, have been highlighted as factors that could facilitate inter-specific hybridization in rabbitfish, the relative importance of hybridization in the diversification of siganids, in general, is currently poorly understood. It is not surprising that most reports of hybridization events have occurred between species pairs that inhabit overlapping geographical regions. Examples include the following species pairs; *S. vulpinus*–*S. unimaculatus* in the Western Pacific region, *S. doliatus*–*S. virgatus* in the Indo-Malay region, and *S. canaliculatus*–*S*. *fuscescens* in the Indo-Australian Archipelago [1,10].

Evidence for introgression has come from biogeographical patterns, the recognition of intermediate morphologies, and small genetic distance estimates as low as 0.2–0.7%. Each member of species-pairs shows only minor morphological differences; specifically, *S. vulpinus* and *S. unimaculatus* can only be differentiated by the presence of black spots on the body, whereas *S. doliatus and S. virgatus* are phenotypically differentiated only by the presence of slender bars or pearly blue spots on the side of the body [1]. The only discernible external difference between *S. fuscescens* and *S. canaliculatus* is the number of body spots, but genetically, they are only 0.2% divergent [1]. In fact, several specimens of *S. fuscescens*–*S. canaliculatus* in the current study showed intermediate morphological and morphometric characteristics resulting from interspecific hybridization. In contrast, Iwamoto et al. [71] proposed another hypothesis, regarding *S. canaliculatus* as a junior synonym of *S. fuscescens* and *S. fuscescens* as a valid species. The two species show similar morphological features with only some limited variation evident in morphological patterns related to an individual’s size: small individuals have sparse spots, whereas larger individuals show more compact spots on their body (Kuriiwa, pers. comm.). When examined closely, both species possess sparse and compact spots in small and large individuals, respectively. A prior study has also suggested that *S. fuscescens* and *S. canaliculatus* display a phenotypic response to external environmental factors that result in variation of these traits among geographically separated populations [71]. Due to their overall morphological similarities, it was suggested that members of each of the three species pairs actually comprise of a single biological species that possesses multiple genetic color morphs [7]. They could not, however, reject the hypothesis that they constituted valid incipient species. Given the differences in interpretations between these two studies, further analysis with samples collected from multiple localities will be needed to reach a definitive conclusion on the taxonomic status of *S. fuscescens* and *S. canaliculatus*. Phylogeographic inference has implicated historical fluctuations in sea levels over the Pliocene/Pleistocene that could have caused vicariate speciation in siganid taxa as a result of local natural selection, whereas modern-day hybridization between siganid taxa most likely results from range expansions that have led to contact and hybridization events between only recently evolved sister taxa.

One of the most remarkable outcomes from the current study is a new record for *S. sutor* in the Indo-Malay region, particularly along the Peninsular Malaysian eastern coastline. Although we have validated this new occurrence after a comprehensive morphological analysis of 10 specimens of *S. sutor* collected in this region, a larger sample size that covers a more extensive distribution is warranted in the future study. Of interest, although *S. sutor* is morphologically similar to both *S. fuscescens* and *S. canaliculatus*, COI sequences could successfully discriminate this species from both latter species. Thus, Borsa et al.’s. [10] suggestion that a common ancestor existed for the sister lineages of *S. fuscescens*–*S. canaliculatus* and *S. sutor*–*S. rivulatus* resulting from allopatric divergence due to historical tectonic activity across the Indonesian region [72,73] can now likely be rejected due to the modern-day presence of *S. sutor* in the Malay Peninsula region.

## 5. Conclusions

The current study provides a robust molecular phylogeny and estimates for the temporal diversification of modern rabbitfish. Based on diagnostic morphological features, morphometric, and molecular phylogenetic analyses, we suggest that the family Siganidae diverged from a common generalized ancestor into three major clades characterized by specific variation in body shape and two morphometric parameters (body depth and snout length). We also postulate that the visual system may play an important role in species diversification in siganids, and the involvement of additional *Siganus* species is warranted in future rhodopsin gene studies. We hypothesize that the most remarkable diversification of siganid lineage occurred at the end of the Miocene extending through to the Pliocene/Pleistocene, coinciding with major paleo-oceanographic changes. Episodes of greenhouse events, tectonic events, global cooling, and associated environmental change likely influenced the origins and diversification of modern siganid lineages. In general, our results emphasize the importance of morphological characteristics, morphometric measurements, and ecological traits in driving pathways of siganid evolution. In future studies, additional taxa sampling that should include a representative of all known siganids comprising 29 extant species that combine morphological and molecular markers will be needed to better resolve the phylogeny, phylogeography, and diversification history of this teleost family.

## Figures and Tables

**Table 1:** Morphometric measurements (as % of standard length) for discrimination of three deep-bodied species collected from the South China Sea, Terengganu, Malaysia.

Species	Morphometric Variables	Mean	SD	Range	Sig. (*p*)	Correlation Coefficient (r)
*S. guttatus*	Snout length	14.1	0.8	12.5–15.5	0.604	0.152
	Length of anal fin base	43.5	1.3	42–46.4	0.788	0.079
	Depth of caudal peduncle	8.1	0.4	7.1–8.9	0.976	0.009
	Length of lower jaw	5.0	0.4	4.4–5.6	0.054	0.407
*S. javus*	Snout length	10.8	0.6	9.8–11.7	0.683	0.097
	Length of anal fin base	47.2	1.0	45.6–49.0	0.042	0.458
	Depth of caudal peduncle	6.8	0.4	6.0–7.4	0.085	0.395
	Length of lower jaw	4.2	0.3	3.7–4.6	0.075	0.072
*S. stellatus*	Snout length	12.4	0.9	10.6–13.8	0.002	0.650
	Length of anal fin base	42.3	1.2	40.1–44.4	0.945	0.016
	Depth of caudal peduncle	7.6	0.4	7.0–8.4	0.402	0.198
	Length of lower jaw	3.9	0.3	3.3–4.3	0.764	0.007

## Data Availability

The fish specimens were stored in the South China Sea Repository and Reference Center, Universiti Malaysia Terengganu. All sequences generated in this study were deposited in GenBank. For details of the GenBank accession number, please see Table S1.

## Supplementary Materials

The following are available online at https://www.mdpi.com/article/10.3390/biology10111109/s1. Table S1. List of individuals sequenced, species descriptions based on main diagnostic morphological features and morphometric parameters. Table S2. Primer sequences used in the study. Table S3. GenBank and BOLD sequence data for all available siganid species of cytochrome oxidase I (COI) from the database. Table S4. Pairwise genetic distance (Tamura-Nei +G model, TN93 + G) among 18 siganid taxa based on COI gene sequences. Table S5. Pairwise genetic distance (Jukes Cantor + G model, JC + G) among 8 siganid taxa based on RHO gene sequences. Figure S1. The illustration on (A) traditional morphometric and (B) geometric measurements. Figure S2. Pairwise comparisons between the three deep-bodied species: *S. guttatus*, *S. javus*, and *S. stellatus*. Figure S3. Maximum Likelihood (ML) phylogenetic relationship between 18 siganid taxa based on concatenated COI and RHO gene sequences.
