# Supplementary material for: Evolutionary History and Taxonomic Reappraisal of Coral Reef Rabbitfishes (Siganidae): Patterns of Lineage Diversification and Speciation
Source: Biology (Basel). 2021 Oct 28;10(11):1109. doi: 10.3390/biology10111109 (PMC8615046; doi:10.3390/biology10111109)
Supplement: Supplementary file 1 [file biology-10-01109-s001.zip › biology-1419130-supplementary.pdf]

Table S1. List of individuals sequenced, species descriptions based on main diagnostic morphological features and morphometric parameters

Species	n	Accession Numbers		Diagnostic Morphological Features					Morphometric Parameters as % SL, range (mean ± std. dev.)		
		COI	Rhodopsin	Color type	Body shape	Caudal fin	Body color	Body Spots/ Bands	Body depth	Snout length	Eye diameter
Siganus argenteus	1	KT997961 (1)	KU182537 (1)	Drab	Moderately slender and compressed	Strongly forked	Yellow (head), deep sea blue (upper part), pale blue (lower part)	Dark spots on top of head	35.0	8.8	7.1
Siganus canaliculatus	25	KT997891–KT997910 (18)	KU182538–KU182555 (18)	Drab	Slender and compressed	Almost emarginated (Young fish) Forked (Adult)	Silvery grey (Upper part), silvery (Lower part)	100–200 pearly blue to whitish spots on nape and trunk	36.1–43.4 (**39.8** ± 1.8)	9.3–11.6 (**10.4** ± 0.5)	6.6–10.2 (**8.1** ± 0.8)
Siganus fuscescens	25	KT943378–KT943398 (20)	KU182556–KU182577 (22)	Drab	Slender and compressed	Almost emarginated (Young fish) Forked (Adult)	Olive green or brown (Upper part), silvery (Lower part)	>180 pearly blue spots on head and trunk	35.3–42.1 (**38.7** ± 1.6)	8.8–11.6 (**10.4** ± 0.7)	6.3–9.2 (**7.7** ± 0.8)
Siganus sutor	10	KT997949; KT997950–KT997958 (10)	KU182630–KU182638 (9)	Drab	Slender and compressed	Forked, yellow brown in color	Greenish grey (Upper part), Paler green (Lower part)	-	36.2–42.6 (**39.1** ± 1.8)	8.9–12.4 (**10.4** ± 1.1)	6.6–10.1 (**8.0** ± 1.2)
Siganus guttatus	14	KT943399–KT943410 (12)	KU182578–KU182590 (13)	Bright	Deep and compressed	Emarginated	Blue (Upper part), silvery (Lower part)	Bright yellow spot on sides at base of last few rays of dorsal fin	44.8–51.0 (**48.1** ± 1.6)	12.5–15.5 (**14.1** ± 0.8)	8.1–10.0 (**9.0** ± 0.6)
Siganus javus	20	KT997912–KT997930 (19)	KU182591–KU182609 (19)	Bright	Deep and compressed	Emarginated, dusky with faint vertical bar	Black and dark bronze (Upper part), paler black-bronze (Lower part)	Gun-metal grey spots on head and upper sides; Pale silvery blue undulating lines on mid and lower sides	45.3–52.3 (**49.1** ± 2.0)	9.8–11.7 (**10.8** ± 0.6)	7.8–12.1 (**9.2** ± 1.1)
Siganus stellatus	20	KT997932–KT997948 (17)	KU182610–KU182629 (20)	Bright	Deep and compressed	Emarginated (Young fish) Forked (Adult)	Greyish green	Brown spots on head and trunk	42.8–47.0 (**44.9** ± 1.2)	10.6–13.8 (**12.4** ± 0.9)	6.1–9.4 (**7.5** ± 0.9)
Siganus virgatus	2	KT997959–KT997960 (2)	KU182639–KU182640 (2)	Bright	Deep and compressed	Emarginated, yellow in color	Lemon yellow (Upper part), silvery with rosy flush (Mid-side of lower part)	Brown band on nape, base of 4th to 6th dorsal spine and base of pectoral fin	48.6–51.0 (**49.8** ± 1.7)	13.2–13.3 (**13.3** ± 0.1)	8.1–9.2 (**8.7** ± 0.8)

Table S2. Primer sequences used in the study

Gene	Primer name	Sequence (5′ to 3′)	Amplicon size (bp)	Annealing temperature (°C)	References
Cytochrome oxidase subunit I (COI)	FF2d<br>FR1d	TTCTCCACCAACCACAARGAYATYGG<br>CACCTCAGGGTGTCCGAARAAYCARAA	655	51.3	Modified protocols from Ivanova et al. (2007)
Rhodopsin retrogen (RHO)	Rod_F2W<br>Rod_R4n	AGCAACTTCCGCTTCGGTGAGAA<br>GGAACTGCTTGTTCATGCAGATGTAGAT	460	60	Modified protocols from Sevilla et al. (2007)

Table S3. GenBank and BOLD sequence data for all available siganid species of cytochrome oxidase I (COI) from the database

No.	Species	Sample size (n)	Locality	BOLD ID (Accession No.)/Accession No.
1.	*S. corallinus*	2	Philippines	EU620486-EU620487 (BOLD: AAC8755)
2.	*S. doliatus*	2	Australia	KP194691; KF930440
3.	*S. luridius*	1	Madagascar	JQ350366
4.	*S. puellus*	2	Sanya (China); Manila (Philippines)	EU620478 (BOLD: AAJ6676); FJ584108
5.	*S. punctatus*	2	N/A	EU620481 (BOLD: AAB2341); KP194265
6.	*S. rivulatus*	7	Israel	KM538560-KM538561; N/A (BOLD: ABY0829)
7.	*S. spinus*	8	French (Polynesia); Philippines	JQ432158-JQ432159 (BOLD: AAE9661); EU620485 (BOLD: ACX8815); EU620484 (BOLD: ACX8816); KCQ59886-KCQ59887; KC970420; KF009664
8.	*S. vermiculatus*	3	Philippines	KF715016-KF715018
9.	*S. (Lo) vulpinus*	9	Guangdong (China)	NC025588 (BOLD: AAD9299); KM233212, EU620480 (BOLD: AAD9299); KF009667; FJ584114-FJ584118
10.	*S.(Lo) unimaculatus*	3	Japan, Philippines	NC013148, AF006031, EU620479 (BOLD: AAD9299)
	**TOTAL**	**39**		

Table S4. Pairwise genetic distance (HKY+G, Hasegawa-Kishino-Yano +G Model) among 18 siganid taxa based on COI gene sequences

	Species	1	2	3	4	5	6	7	8	9	10	11	12	13	14	15	16	17	18
**1**	***S. argenteus***		0.026	0.025	0.024	0.026	0.025	0.027	0.028	0.029	0.027	0.030	0.030	0.027	0.035	0.029	0.027	0.023	0.028
**2**	***S. canaliculatus***	0.165		0.030	0.029	0.002	0.028	0.028	0.013	0.031	0.029	0.015	0.020	0.029	0.017	0.027	0.026	0.028	0.027
**3**	***S. corallinus***	0.162	0.202		0.007	0.030	0.013	0.021	0.030	0.022	0.019	0.033	0.029	0.019	0.032	0.018	0.012	0.006	0.017
**4**	***S. doliatus***	0.148	0.188	0.019		0.029	0.012	0.019	0.027	0.020	0.019	0.031	0.026	0.019	0.030	0.018	0.011	0.004	0.017
**5**	***S. fuscescens***	0.169	0.002	0.202	0.188		0.028	0.028	0.013	0.031	0.029	0.015	0.020	0.029	0.017	0.027	0.026	0.028	0.027
**6**	***S. guttatus***	0.159	0.184	0.056	0.051	0.184		0.021	0.029	0.019	0.018	0.028	0.028	0.019	0.032	0.018	0.012	0.011	0.018
**7**	***S. javus***	0.176	0.178	0.119	0.104	0.178	0.111		0.024	0.015	0.016	0.026	0.029	0.016	0.028	0.017	0.019	0.019	0.017
**8**	***S. luridus***	0.174	0.061	0.195	0.171	0.058	0.186	0.149		0.025	0.027	0.012	0.018	0.027	0.012	0.026	0.025	0.027	0.026
**9**	***S. puellus***	0.192	0.203	0.125	0.110	0.203	0.105	0.072	0.164		0.015	0.026	0.028	0.015	0.027	0.017	0.020	0.020	0.016
**10**	***S. punctatus***	0.166	0.180	0.106	0.103	0.180	0.099	0.078	0.167	0.075		0.027	0.028	0.002	0.029	0.013	0.019	0.019	0.012
**11**	***S. rivulatus***	0.187	0.075	0.220	0.202	0.072	0.184	0.162	0.049	0.173	0.177		0.020	0.028	0.013	0.026	0.027	0.031	0.026
**12**	***S. spinus***	0.200	0.123	0.193	0.169	0.123	0.186	0.182	0.099	0.181	0.182	0.124		0.029	0.020	0.031	0.028	0.027	0.030
**13**	***S. stellatus***	0.169	0.183	0.103	0.106	0.183	0.102	0.082	0.170	0.078	0.002	0.180	0.185		0.029	0.013	0.019	0.019	0.011
**14**	***S. sutor***	0.220	0.094	0.204	0.192	0.091	0.203	0.179	0.050	0.179	0.190	0.058	0.124	0.186		0.028	0.025	0.030	0.028
**15**	***S. unimaculatus***	0.188	0.181	0.098	0.102	0.181	0.098	0.093	0.174	0.087	0.059	0.177	0.212	0.056	0.186		0.018	0.017	0.004
**16**	***S. vermiculatus***	0.168	0.168	0.048	0.043	0.168	0.050	0.101	0.161	0.109	0.106	0.176	0.183	0.109	0.158	0.098		0.010	0.018
**17**	***S. virgatus***	0.145	0.181	0.016	0.007	0.181	0.048	0.104	0.171	0.113	0.100	0.195	0.179	0.103	0.186	0.095	0.040		0.017
**18**	***S. vulpinus***	0.185	0.178	0.090	0.093	0.178	0.096	0.090	0.171	0.078	0.051	0.173	0.205	0.048	0.182	0.007	0.096	0.087	

S. = *Siganus*; Standard error estimate(s) are shown above the diagonal

Table S5. Pairwise genetic distance (Hasegawa-Kishino-Yano +G Model) among 8 siganid taxa based on RHO gene sequences

	Species	1	2	3	4	5	6	7	8
**1**	***S. argenteus***		0.011	0.011	0.010	0.010	0.011	0.011	0.010
**2**	***S. canaliculatus***	0.046		0.000	0.009	0.009	0.009	0.000	0.009
**3**	***S. fuscescens***	0.046	0.000		0.009	0.009	0.009	0.000	0.009
**4**	***S. guttatus***	0.044	0.034	0.034		0.004	0.005	0.009	0.000
**5**	***S. javus***	0.041	0.036	0.036	0.007		0.003	0.009	0.004
**6**	***S. stellatus***	0.046	0.036	0.036	0.011	0.004		0.009	0.005
**7**	***S. sutor***	0.046	0.000	0.000	0.034	0.036	0.036		0.009
**8**	***S. virgatus***	0.044	0.034	0.034	0.000	0.007	0.011	0.034	

S. = *Siganus*; Standard error estimate(s) are shown above the diagonal

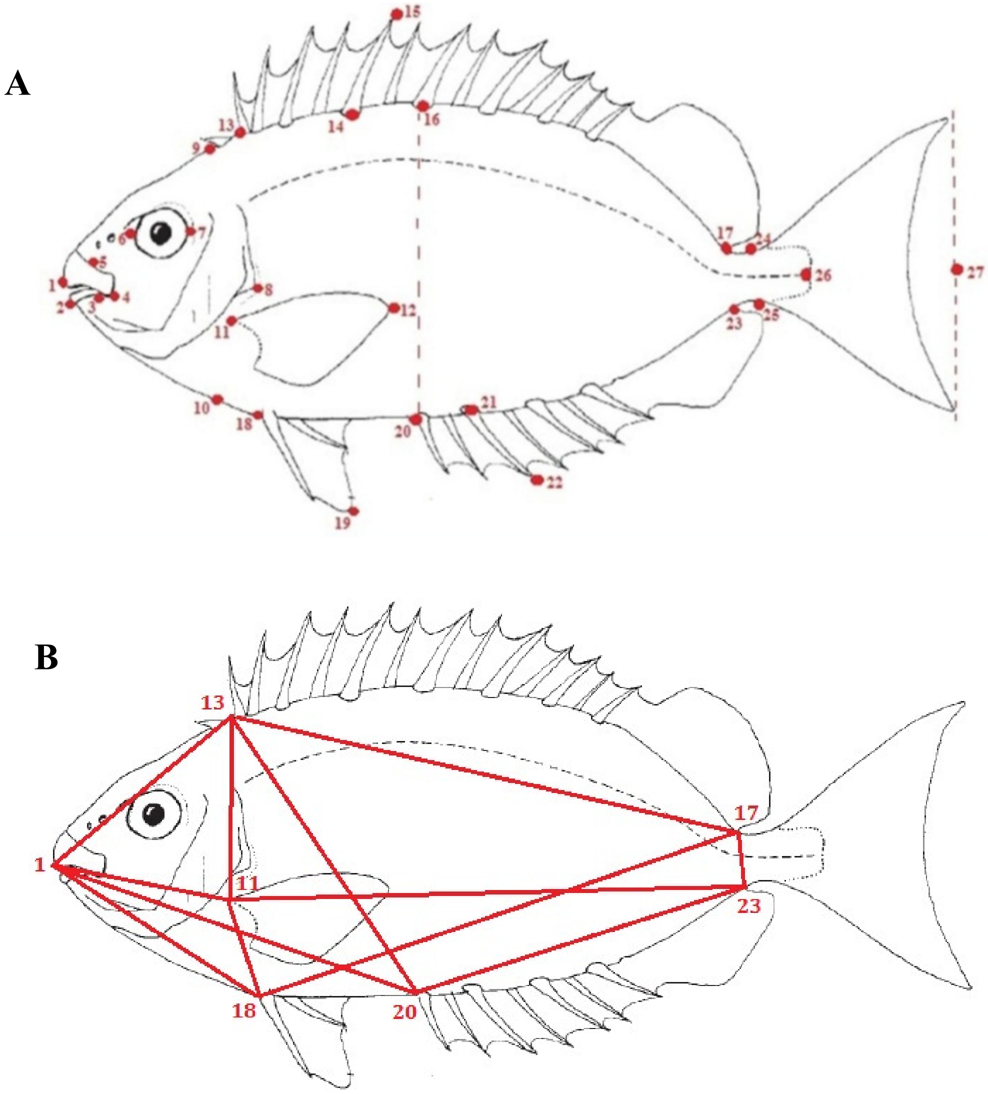

Figure S1. The illustration on (A) traditional morphometric and (B) geometric measurements. Coordinates are indicated by dots and numbers. Description on traditional morphometric and geometric characteristics are presented in next page.

Landmarks	Traditional Morphometric
1-27	Total length
1-26	Standard length
1-8	Head length
9-10	Head depth (at the edge of pre-opercle)
13-18	Head depth (at the edge of opercle)
1-6	Snout length
5-6	Suborbital depth
1-4	Length of upper jaw
2-3	Length of lower jaw
6-7	Orbit diameter
7-8	Post-orbital length
1-11	Pre-pectoral length
1-18	Pre-pelvic length
1-20	Pre-anal length
1-13	Pre-dorsal length
13-17	Length of dorsal fin base
14-15	Height of dorsal fin
11-12	Length of pectoral fin
18-19	Length of pelvic fin
20-23	Length of anal fin base
21-22	Height of anal fin
23-26	Length of caudal peduncle
24-25	Depth of caudal peduncle
16-20	Body depth
	**Geometric**
11-18	Length between insertion of P1 and insertion of P2
17-18	Length between end of D and insertion of P2
13-20	Length between origin of D and origin of A
11-23	Length between insertion of P1 and end of A
18-20	Length between insertion of P2 and origin of A
17-23	Length between end of D and end of A

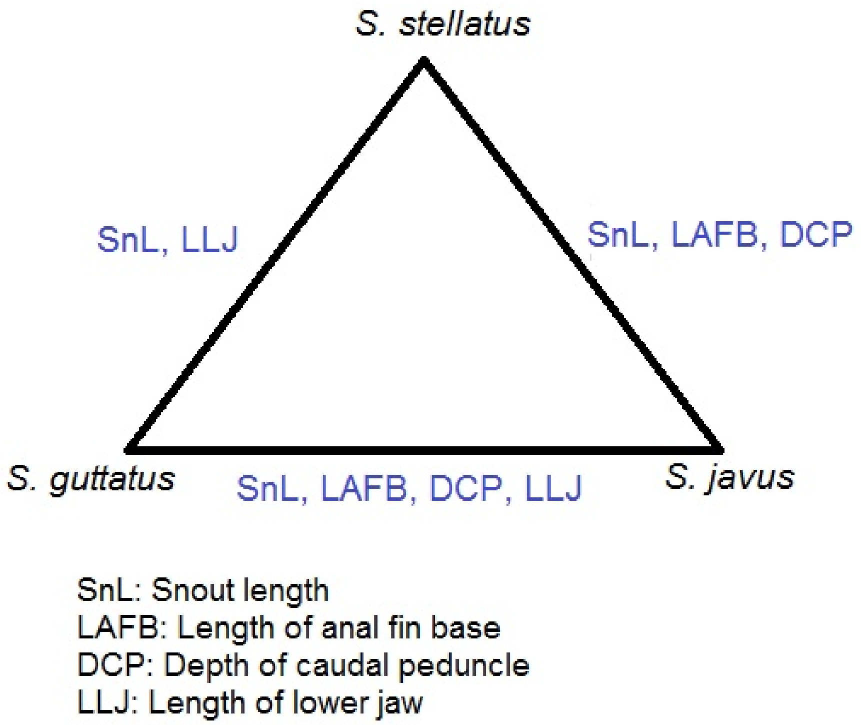

Figure S2. Pairwise comparisons between the three deep-bodied species: *S. guttatus*, *S. javus* and *S. stellatus*.

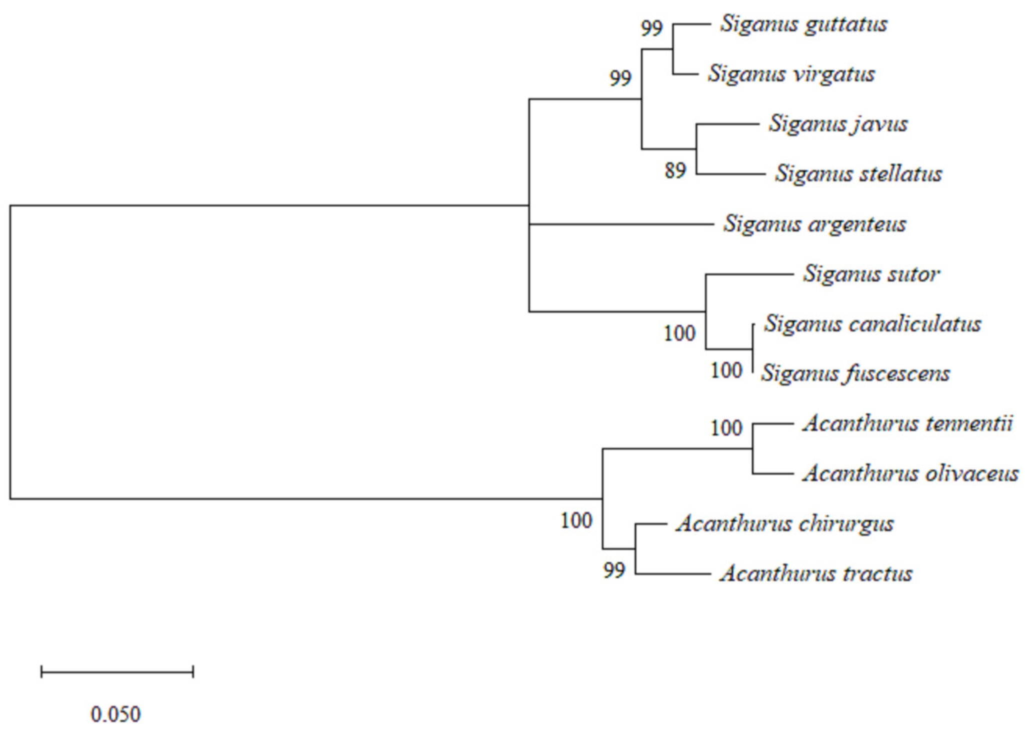

Figure S3. Maximum Likelihood (ML) phylogenetic relationship between 8 siganid taxa based on concatenated COI and RHO gene sequences. Bootstrap values are shown next to the branches, and scale bar represents branch lengths measured in the number of substitutions per site.
